# Regulation of *in vitro* human T cell development through interleukin-7 deprivation and anti-CD3 stimulation

**DOI:** 10.1186/1471-2172-13-46

**Published:** 2012-08-16

**Authors:** Ekta S Patel, Starlyn Okada, Kevin Hachey, Li-jun Yang, Scott K Durum, Jan S Moreb, Lung-Ji Chang

**Affiliations:** 1 Department of Molecular Genetics and Microbiology, College of Medicine, University of Florida, Gainesville, FL, 32610, USA; 2 Department of Pathology, Immunology and Laboratory Medicine, College of Medicine, University of Florida, Gainesville, FL, 32610, USA; 3 Section of Cytokines and Immunity, National Cancer Institute, Frederick, MD, 32610, USA; 4 Department of Medicine, College of Medicine, University of Florida, Gainesville, FL, 32610, USA; 5 Department of Molecular Genetics and Microbiology, University of Florida, 1600 SW Archer Road, ARB R1-252, Gainesville, FL, 32610, USA

**Keywords:** T cell development, Interleukin-7, T cell receptor, Vbeta repertoire

## Abstract

**Background:**

The role of IL-7 and pre-TCR signaling during T cell development has been well characterized in murine but not in human system. We and others have reported that human BM hematopoietic progenitor cells (HPCs) display poor proliferation, inefficient double negative (DN) to double positive (DP) transition and no functional maturation in the *in vitro* OP9-Delta-like 1 (DL1) culture system.

**Results:**

In this study, we investigated the importance of optimal IL-7 and pre-TCR signaling during adult human T cell development. Using a modified OP9-DL1 culture ectopically expressing IL-7 and Fms-like tyrosine kinase 3 ligand (Flt3L), we demonstrated enhanced T cell precursor expansion. IL-7 removal at various time points during T cell development promoted a slight increase of DP cells; however, these cells did not differentiate further and underwent cell death. As pre-TCR signaling rescues DN cells from programmed cell death, we treated the culture with anti-CD3 antibody. Upon pre-TCR stimulation, the IL-7 deprived T precursors differentiated into CD3^+^TCRαβ^+^DP cells and further matured into functional CD4 T cells, albeit displayed a skewed TCR Vβ repertoire.

**Conclusions:**

Our study establishes for the first time a critical control for differentiation and maturation of adult human T cells from HPCs by concomitant regulation of IL-7 and pre-TCR signaling.

## Background

Generation of mature human T cells from adult bone marrow (BM) CD34^+^HPCs *in vitro* may overcome two major limitations in T cell therapy, namely HLA disparity and immune tolerance. Patients undergoing chemotherapy or with HIV infection suffer from prolonged lymphodepletion leading to opportunistic infections and mortality [1]. Hematopoietic stem cell transplant (HSCT) has been used to reconstitute the immune system in such patients [2]. However, T cells take the longest time to recover after HSCT [2]. Thus *ex vivo* differentiation of T cells using an *in vitro* OP9 stromal cell line expressing Notch ligand, Delta like-1 (DL1), has been of tremendous interest [3-5]. Recent reports showed that the OP9DL1 stromal cell culture system established by Zuniga-Pflucker can support terminal maturation of cord blood (CB) and post natal thymus derived CD34^+^HPCs [6,7]. In case of immune rejection of CB HPCs due to HLA disparity or limited availability, BM CD34^+^HPCs may serve as a convenient source as they can be obtained from patient’s own BM [8,9]. We and others have demonstrated that adult BM-derived CD34^+^HPCs, from both normal adults and patients undergoing chemotherapy, yields a low number of T cell precursors *in vitro*[10-12]. T cell development of adult human BM derived CD34^+^HPCs in the OP9 DL1 culture system is less well studied due to low cellular yields when compared to the CB counterparts. In addition, terminal T cell differentiation from adult human BM derived CD34^+^HPCs *in vitro* has not yet been demonstrated [10,13].

We have previously reported that lentivector-modified OP9 cell lines expressing various cytokines and growth factors supported enhanced HPC and dendritic cell precursor expansion and differentiation [14]. To overcome the limited proliferation of BM HPCs *in vitro*, we modified a previously defined LmDL1 cell line (Lentivector-modified OP9 expressing DL1) [10], to ectopically express T cell developmental factors IL-7 and Flt3L, and established LmDL1-FL7 cell line. We found that LmDL1-FL7 provided a proliferative advantage to adult BM CD34^+^HPCs over LmDL1 cell line supplemented with soluble recombinant hIL-7 and hFlt3L.

During T cell development, the CD34^+^CD8^-^CD4^-^ double negative (DN) thymocytes differentiate through CD3^-^CD8^+^ immature single positive stage (ISP) in mice and CD3^-^CD4^+^ ISP in humans, followed by CD3^lo^CD4^+^CD8^+^ double positive (DP), CD3^+^TCRαβ^+^DP and then CD3^+^TCRαβ^+^CD4^+^ or CD8^+^ mature single positive T cells [15,16]. We observed that the transition of CD3^lo^ DP to CD3^+^TCRαβ^+^ DP stage, an intermediate stage that precedes the terminal maturation to CD8 or CD4 T cell lineage, is inefficient during adult BM T cell development *in vitro*[17]. IL-7 plays an inhibitory role during DN to DP transition in mice [18-22] and signaling via CD3/Pre-TCR complex plays a permissive role in transition from CD3^lo^ DP to CD3^+^ TCRαβ^+^ DP [23-25]. Thus, we hypothesized that the inefficient pre-TCR signaling is either due to continued presence of IL-7 or due to inefficient stimulation via CD3 receptor. Here we report that intermittent IL-7 withdrawal alone did not result in efficient differentiation to CD3^+^TCRαβ^+^ DP stage. Importantly, taking a combination approach of IL-7 withdrawal and activating pre-TCR signaling using anti-CD3/CD28 antibodies, we demonstrate for the first time *in vitro* differentiation of adult BM HPCs to CD3^+^TCRαβ^+^ DP stage and subsequent functional maturation of CD4 T cells. Our findings provide a better understanding of the factors involved in proliferation and differentiation of BM derived HPCs to mature T cells *in vitro*.

## Results

### OP9-DL1 cells ectopically expressing Flt3L and IL-7 support enhanced T cell precursor expansion

**Figure 1:**
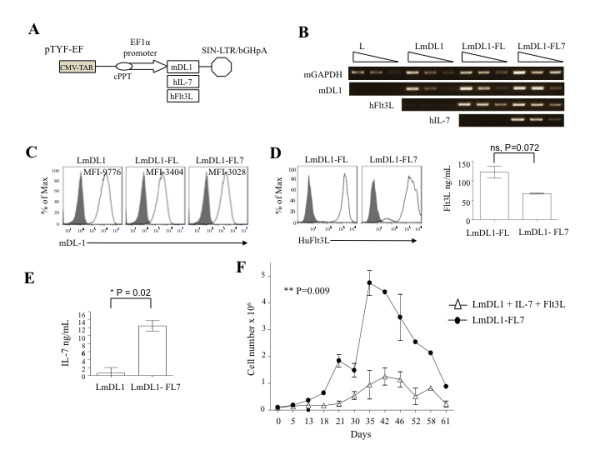
**Enhanced proliferation of T cell precursors on LmDL1-FL7.*A***, Lentivector constructs expressing mouse DL1, human IL-7 and human Flt3L. ***B***, qRT-PCR analysis for DL-1, Flt3L, and IL-7. ***C*** &***D***, Flow cytometry analysis of mDL-1 (***C***) and Flt3L (***D***) surface expression in the various OP9 cell lines. ELISA analyses for Flt3L (***D***) and IL-7 (***E***) production. ***F***, Growth kinetics of developing T cells from adult human BM-derived CD34^+^ HPCs on LmDL + IL-7 + Flt3L versus LmDL1-FL7 with p value indicated.

The previously established mouse OP9-DL1 cell line, LmDL1 [10], was infected with lentivectors expressing human Flt3L, or both Flt3L and IL-7, to generate LmDL1-FL and LmDL1-FL7 cell lines, respectively (Figure 1A). RNA was harvested and analyzed by semi-quantitative RT-PCR to confirm transgene expression in these cell lines (Figure 1B). We confirmed surface expression of DL1 on all three cell lines, LmDL1, LmDL1-FL and LmDL1-FL7 (Figure 1C). Both LmDL1-FL and LmDL1-FL7 expressed high levels of Flt3L on cell surface and in culture as determined by flow cytometry and ELISA, respectively (Figure 1D). The secretion of IL-7 by LmDL1-FL7 was measured via ELISA to be in the range of 10–14 ng/mL after 48 hr of culture (Figure 1E). 

To examine the differentiation and expansion potential of adult human BM CD34^+^ HPCs co-cultured with LmDL1 exogenously supplemented with recombinant human Flt3L (5 ng/mL) and IL-7 (5 ng/mL), or co-cultured with LmDL1-FL7, we determined the proliferation rate of the incubated cells by counting total number of suspension cells at various time points in three independent experiments. The result showed that CD34^+^ HPCs cells, when co-cultured with LmDL1-FL7 for 35 days, expanded up to five fold more than those co-cultured with LmDL1 supplemented with recombinant Flt3L and IL-7 (Figure 1F, representative of three donors). Thus, LmDL1-FL7 was superior to LmDL1 in supporting T cell precursor proliferation.

### Adult BM CD34^+^ HPCs co-cultured on LmDL1-FL7 or LmDL1 supplemented with IL-7 and Flt3L follow similar T cell differentiation kinetics but do not undergo functional T cell maturation

**Figure 2:**
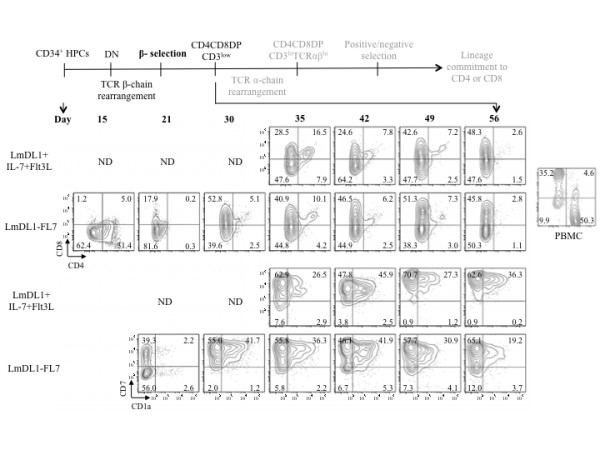
**Flow cytometry analyses of expression kinetics of CD8, CD4, CD7, and CD1a of the developing T cells under LmDL1 + IL-7 + Flt3L or LmDL1-FL7 co-culture condition.** Schematic illustration of key stages of T cell development is shown at top. Adult human BM CD34^+^ HPCs were plated on LmDL1-FL7 or LmDL1 + IL-7 + Flt3L and surface expression of CD8, CD4, CD7 and CD1a was examined over time as depicted. The percentages of stained cells are indicated in the flow graph quadrons. ND, not done, due to insufficient amount of developing T cells from the early time points of the LmDL1 + IL-7 + Flt3L coculture.

**Figure 3:**
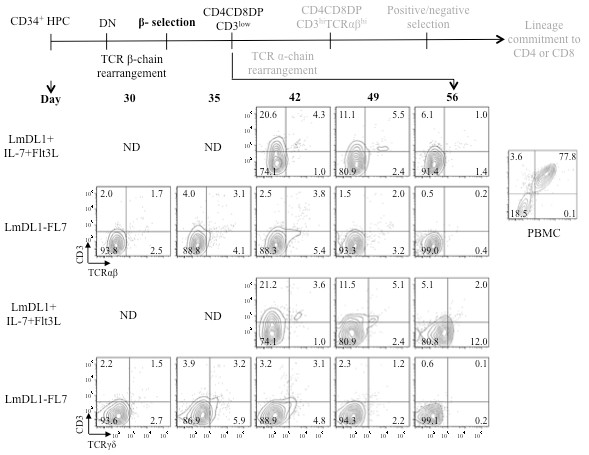
**Flow cytometry analyses of expression kinetics of CD3, TCRαβ and TCRγδ of the developing T cells under LmDL1 + IL-7 + Flt3L or LmDL1-FL7 co-culture condition.** Schematic illustration of key stages of T cell development is shown at top. T cell markers CD3, TCRαβ, and TCRγδ were analyzed for the developing T cells under the two different co-culture conditions up to 56 days. ND, not done, due to insufficient amount of developing T cells from the early time points of the LmDL1 + IL-7 + Flt3L coculture.

Next, we analyzed surface expression of CD8, CD4, CD7, CD1a, CD3, TCRαβ and TCRγδ of the differentiating cells in the two co-culture systems. Kinetics of CD8, CD4, CD7 and CD1a were comparable between the two systems. We observed CD4 ISP from day 5 (not shown) to day 15, and increased CD8 ISP after day 20 (Figure 2). T cell lineage commitment from HPCs is defined by upregulation of CD7, followed by CD1a expression which is decreased upon further maturation. A schematic illustration of the predicted key events and phenotypes of developing T cell precursors is shown at top of Figures 2 and 3. We detected surface CD7 on day 15 (data not shown), and peaked expression of CD7 and CD1a around day 42, followed by a gradual decrease of CD1a. Due to the low cell number in the LmDL1 + IL-7 + Flt3L coculture, we had limited cells for analysis in the early time points. In both systems, DP cells appeared around day 35 and decreased by day 56. The timing of appearance of CD8 ISP and DP varied depending on the donor, and was similar between the two systems. The rapid expansion of T cell precursors in co-culture with LmDL1-FL7, which continuously produced Flt3L and IL-7, was accompanied by a slower transition into DP and CD3 positive stage, as both DP cells and CD3 surface expression were detected at lower levels in LmDL1-FL7 co-culture than in LmDL1 + IL-7 + Flt3L coculture (Figures 2 &3). Nevertheless, neither system produced CD3^hi^TCR^hi^ CD4 or CD8 cell population (Figure 3). The analysis of TCRγδ surface markers detected no γδ lineage diversification in LmDL1-FL7 co-culture but some in LmDL1 + IL-7 + Flt3L (Figure 3 bottom). Thus, we conclude that no functionally mature T cells could be generated from the adult BM-derived CD34^+^ HPCs in the *in vitro* cultures.

### IL-7 deprivation alone does not induce efficient DN to DP transition

**Figure 4:**
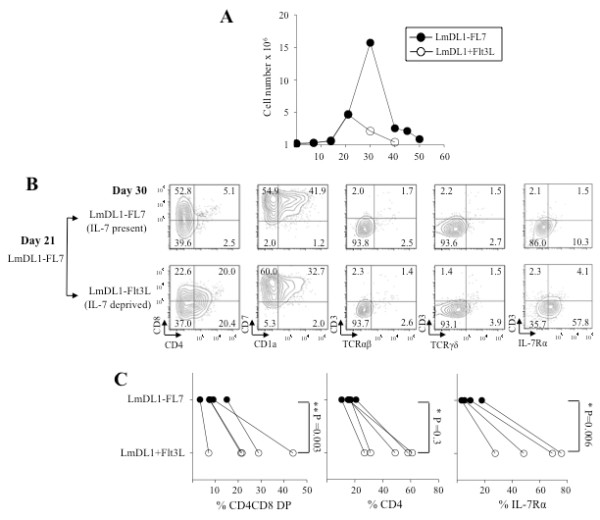
**The effect of IL-7 withdrawal on T cell differentiation.** Adult BM CD34^+^ HPCs were co-cultured on LmDL1-FL7 for 21 days and continued on LmDL-FL7 (IL-7 present) or transferred to LmDL-Flt3L (IL-7 deprived) for additional nine days. ***A***, Growth curves for the developing T cells in the presence or absence of IL-7 after day 21. The cell growth declined markedly upon IL-7 removal at day 21 (depicted by open circles, data represents 4 independent experiments). ***B***, Analysis of T cell markers in the T cell development cultures with or without IL-7 withdrawal after day 21. ***C***, Summary of flow cytometry analysis of surface marker CD4CD8DP, CD4, and IL-7Ra in the developing T cells with (LmDL1-FL7) or without IL-7 (LmDL1-Flt3L), and p values were determined as shown.

In murine T cell development, IL-7 plays a negative role during transition of DN to DP T cells [18-22]. Human thymocytes have been reported to lose IL-7 dependency upon reaching CD7/CD1a DP stage [20]. We found that day 21 cells were always negative for CD1a expression; hence we chose this time point for IL-7 deprivation to ensure that the cells were in IL-7 dependent phase of development. To assess the effect of IL-7 deprivation on DN to DP transition, we cultured adult CD34^+^ HPCs on LmDL1-FL7 for 21 days, followed by continued presence (IL-7 present) or deprivation (IL-7 deprived) of IL-7, i.e., on LmDL1-FL7 or LmDL1-Flt3L, for additional 10–15 days and analyzed the expression of CD7, CD1a, CD4, CD8, CD3, TCRαβ and TCRγδ. We observed a decline in cell survival post IL-7 withdrawal indicative of IL-7 dependence (Figure 4A, open circles). We detected an increase in IL-7Ra expression upon IL-7 withdrawal (LmDL1-Flt3L or LmDL1 +Flt3L, IL-7 deprived, Figure 4 B, C, far right). Additionally, we observed an increase in percentage of DP cells; however, these cells lacked CD3^hi^ TCRαβ^hi^ phenotype (Figure 4 B, C). Thus, IL-7 deprivation alone is not sufficient to induce CD3^hi^ TCRαβ^hi^ DP transition of adult human T cell precursors on the OP9 culture system. 

### IL-7 withdrawal does not increase T cell receptor excision circle (TREC) in the developing T cell precursors

**Figure 5:**
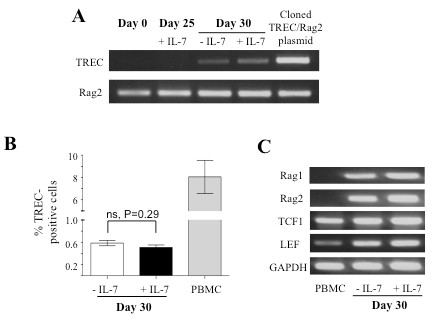
**TCR rearrangement in developing T cell precursors in LmDL1-FL7 co-culture.*A***, TREC analysis in developing T cells. PCR analysis for TREC and Rag2 was performed using genomic DNA harvested at the indicated time points from co-cultures under different IL-7 conditions. ***B***, Quantitative PCR analysis of TREC-positive cells in developing T cells. SYBR Green quantitative PCR analysis of TREC was performed using genomic DNA isolated from T cell precursors under indicated culture conditions. ***C***, Analyses of Rag1, Rag2, TCF1 and LEF RNA expression. The expression of the indicated genes was detected by RT-PCR using 1ug of mRNA harvested from day 30 of the developing T cell precursors.

During T cell development, DN T cell precursors rearrange their TCR beta chain first, which is expressed with pre-TCR alpha to form a pre-TCR complex [26]. Signaling via Pre-TCR complex results in allelic exclusion at TCRβ locus, but initiates rearrangement at the TCRα locus and promotes DP transition [27]. Rearrangement at the TCRα locus can be evaluated by the presence of TREC, an episomal circular piece of DNA formed due to excision of delta locus upon TCRα rearrangement [28]. In order to assess the frequency of αβ precursors, we analyzed TREC content in the developing T cell precursors by genomic PCR. The results showed that cells from day 0 and day 25 were negative, but from day 30 were positive for TREC (Figure 5A). We quantified TREC via quantitative PCR analysis using cloned TREC and RAG2 as standards (Additional file 1) [29]. Our results showed that < 1% cells were positive for TREC on Day 30, and IL-7 deprivation had no effect on TREC content (Figure 5B). RT-PCR analysis of RNAs showed that this was not due to the lack of RAG or TCF1/LEF gene expression (Figure 5C). This result suggests that only a small percent of cells underwent rearrangement at TCRα locus in the *in vitro* system. 

### Adult human HPCs can differentiate to DP T cells and adopt a CD4 T cell lineage *in vitro* upon IL-7 deprivation followed by anti-CD3 stimulation

**Figure 6:**
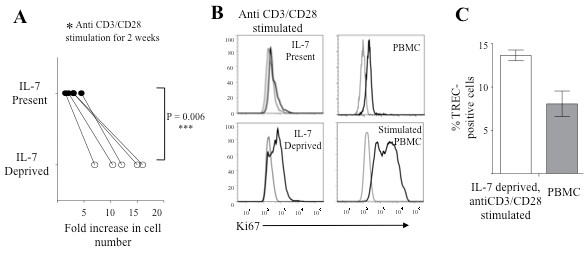
**Increased proliferation and TCR rearrangement of developing T cells upon concomitant IL-7 removal and anti-CD3/CD28 co-stimulation.** Adult human HPCs were co-cultured on LmDL1-FL7 for 21 days and continued on LmDL-FL7 (IL-7 present) or transferred to LmDL-Flt3 (IL-7 deprived) for an additional nine days. ***A***, Diminished precursor T cell growth upon continued presence of IL-7. Day 21 T precursor cells under IL-7 present or deprived conditions were stimulated using anti-CD3/CD28 beads for an additional two weeks. The fold increases in cell number were determined and significant difference was observed between the two groups (n =5, P = 0.006). ***B***, Intracellular staining for Ki67 of the developing T cells with or without IL-7 and stimulated by anti-CD3/CD28 Ab beads. Un-stimulated and stimulated PBMCs from healthy donors were included as controls. ***C***, Quantitative PCR analysis of TREC positive cells in the developing T cell population after IL-7 deprivation and anti-CD3/28 co-stimulation, as compared with control PBMC from healthy donors.

Signaling via pre-TCR complex, composed of TCRβ, pre-TCRα and CD3 is crucial for αβ T cell development [27,30]. Pre-TCR is thought to signal in a ligand independent fashion, possibly through oligomerization [31,32]. Pre-TCR signaling can be mimicked by anti-CD3 antibody stimulation, as *in vivo* administration of anti-CD3 antibody induces DN to DP transition in Rag2^−/−^ pre-Tα^−/−^ mice [25,33]. Also, treatment of fetal thymus organ culture (FTOC) from TCRβ^−/−^, Rag2^−/−^ or SCID mice with anti-CD3 antibodies induces DN to DP transition [24,25]. Thus, we tested if anti-CD3 stimulation of T cell precursors obtained from LmDL co-cultures can induce differentiation to DP stage. In order to maximize cell-cell contact for efficient stimulation, we transferred cells of both IL-7 present and IL-7 deprived groups to U bottom 96 well plates, in a stromal cell free environment and supplemented with anti-CD3/CD28 antibody-conjugated beads. We found that IL-7 deprived precursors proliferated upon anti-CD3 engagement but IL-7 present group did not, as demonstrated by the fold increase in cell number and intracellular Ki67 staining (Figure 6 A, B). On the other hand, TCR activation of the IL-7 present group did not induce proliferation. Additionally, percentage of TREC positive cells increased to ~13% in the IL-7 deprived, anti-CD3 stimulated group of cells, indicating increased TCRα rearrangement (Figure 6C). 

**Figure 7:**
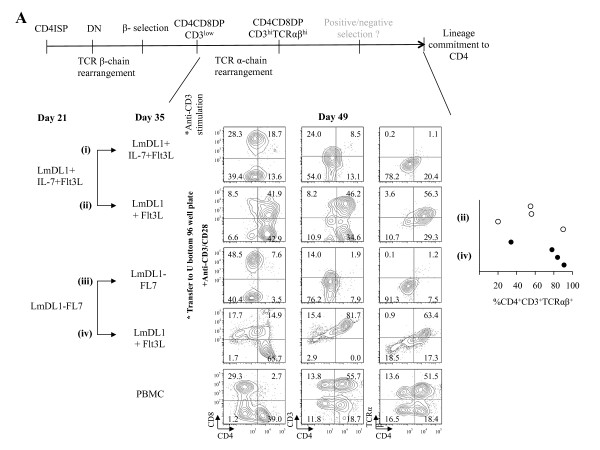
***In vitro*maturation and functional analysis of CD4 SP T cells upon IL-7 deprivation and anti-CD3/CD28 co-stimulation in the absence of stromal cell environment.*A***, Analysis of T cell development kinetics upon IL-7 removal. Diagram of key events and markers for precursor T cell development is illustrated at top. Adult HPCs were co-cultured with stromal cells for 21 days and continued on with IL-7 (IL-7 present, i and iii) or transferred to culture without IL-7 (IL-7 deprived, ii and iv) for an additional 17 days. The cells were then transferred to 96 well U bottom plates and stimulated with anti-CD3/CD28 beads for an additional 14 days. Representative results of analysis of T cell surface markers two weeks after stimulation are shown with percentage of cells indicated in the flow graph quadrons. The percentages of CD4^+^CD3^+^TCRαβ^+^ cell population of four independent T cell development experiments are shown. ***B***, Analysis of effector functions of the *in vitro*-derived CD4 T cells. PBMCs of healthy donors and the *in vitro*-derived CD4 T cells were stimulated using anti-CD3/CD28 beads in the presence of IL-2, IL-7 and IL-15 for two weeks. Expression of intracellular effector cytokine (IFNγ) and T helper functional markers (IL-4, IL-17) was detected after Brefeldin A treatment; unstimulated (left panel) or PMA and ionomycin stimulated (right panel) cells were analyzed by antibody staining and flow cytometry. Note that the small percentage of *in vitro*-derived CD8^+^ cells were not CD3^+^ or viable propagating cells.

We next examined T cell maturation markers 2 weeks post stimulation after deprivation of IL-7. We observed low CD3 expression and no TCRαβ expression in IL-7 present and anti-CD3 stimulated group (Figure 7A, i & iii). Interestingly, anti-CD3 stimulated cells from the IL-7 deprived group displayed a robust transition from CD3^lo^TCRαβ^lo^DP to CD3^+^TCRαβ^+^DP and CD4^+^ SP T cells (Figure 7, ii & iv). Additionally, we found that the cells were mostly negative for CD56 NK cell marker expression (Additional file 2). The timing of IL-7 deprivation and anti-CD3 stimulation was critical, as IL-7 deprivation post day 35 and subsequent anti-CD3 simulation did not induce T cell differentiation and maturation (data not shown). Thus, we conclude that IL-7 deprivation is necessary but not sufficient to promote DP transition and subsequent anti-CD3 stimulation plays a critical role in T cell maturation. To see if the *in vitro* developed CD4 T cells were functional T cells, we further assessed effector functions by IFN-γ, IL-17 and IL-4 secretion in response to PMA and ionomycin. The results showed that upon stimulation, the *in vitro* derived CD4 T cells displayed effector T cell functions similar to that of peripheral blood CD4 T cells (Figure 7B).

### Vβ repertoire analysis of the *in vitro* generated CD4 T cells

**Figure 8:**
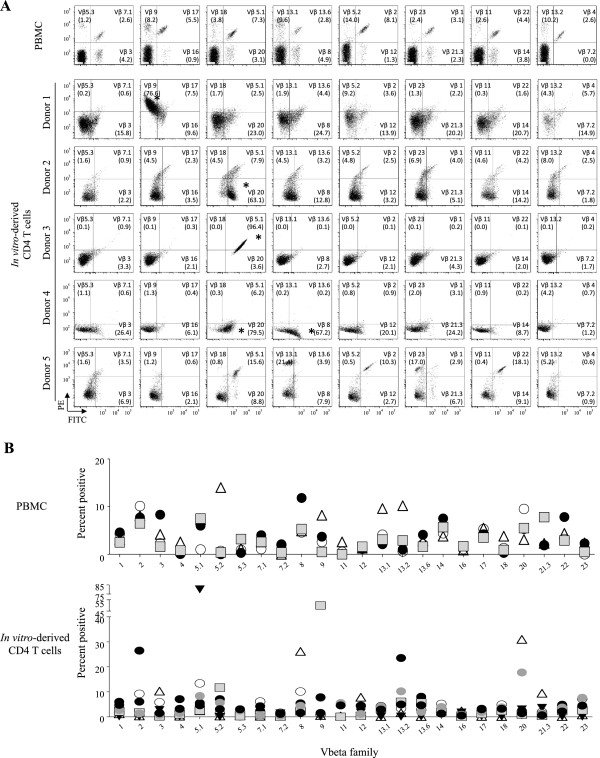
**Vβ repertoire analysis of the*in vitro*-derived CD4 T cells.** After anti-CD3/28 stimulation, the *in vitro-*derived CD4 T cells were surface stained for 24 Vβ families using the IOTest® Beta Mark TCR Vβ Repertoire Kit. CD3-gated population was evaluated for the expression of Vβ families of protein. ***A***, TCR Vβ analysis of *in vitro* developed CD4 T cells of five CD34^+^ HPC donors versus a control PBMC. ***B***, Summary analysis of Vβ distributions of four control PBMCs versus five *in vitro* derived CD4 T cells.

To evaluate the TCR diversity of the *in vitro*-derived T lymphocytes, we performed Vβ repertoire analysis for 23 Vβ families of human TCR. The *in vitro* derived CD4^+^ SP T cells were stained with the IOTest® panel of antibodies. The majority of the *in vitro* differentiated T cells from adult BM HPCs (four of five different donors) displayed a skewed Vβ distribution pattern, e.g. increased populations of Vβ 13.2, 9, 20, 5.1, and 8, respectively, (Donors 1 to 4, marked by * in Figure 8A) as compared with the control PBMCs, which showed an evenly distributed pattern. Note that one of the *in vitro* derived T cells, donor 5, showed a less skewed Vβ distribution pattern. The quantitative analysis of multiple samples is summarized in Figure 8B. We further examined Vβ distributions of healthy donor PBMCs stimulated by anti-CD3/CD28 Ab or PHA for three weeks, and demonstrated that the entire CD4 Vβ clones were evenly expanded without substantial skewing (not shown). Together, we found that the Vβ repertoires of the *in vitro*-derived CD4 T lymphocytes were highly restricted compared with those of normal adult CD4 T cells.

## Discussion

*In vitro* adult human BM HPC-derived functional T cells have great potential for therapeutic applications, as this approach provides donor HLA-matched T cells that may be genetically engineered to fight infections, cancer or to treat immunodeficiencies. Murine HPCs, human CB and post-natal thymic HPCs undergo full maturation in the OP9-DL1 culture system [34,35]. However, adult human BM HPCs undergo limited proliferation and are arrested at CD3^lo^CD4^+^CD8^+^ DP stage of T cell development [10,13]. In this report, we demonstrated that cell-based IL-7 and Flt3L provided a proliferative advantage to adult BM HPCs over exogenously supplemented recombinant IL-7 and Flt3L. We then took a combination approach of IL-7 withdrawal and activating pre-TCR signaling using anti-CD3/CD28 antibodies, which successfully overcame the arrest in CD3^lo^CD4^+^CD8^+^ DP stage and induced differentiation to CD3^+^TCRαβ^+^CD4^+^CD8^+^ DP stage, and subsequent maturation to CD4 T cells. Our findings provide a better understanding of the factors involved in proliferation and differentiation of adult BM-derived HPCs *in vitro*.

LmDL1-FL7 was superior in supporting T cell precursor proliferation when compared to LmDL1 supplemented with exogenous Flt3L and IL-7. The mechanism that enhanced precursor proliferation on LmDL1-FL7 remains to be elucidated. It is possible that concentration or cell-based modifications, or both, could contribute to the enhanced proliferation. As all three cell lines, LmDL1, LmDL1-FL and LmDL1-FL7 expressed high levels of mDL1 (Figure 1), differential DL1 expression level does not seem to play a role. Flt3L is expressed as a soluble as well as membrane bound form [36], and glycosylated form of IL-7 has been reported [37]. Besides soluble factors, cell-cell interactions play a critical role in T cell development. Our results appear to point to the importance of cell-based modification of cytokines, as use of glycosylated IL-7 for clinical trials is being considered [38]. Previous studies demonstrated that a high dose of IL-7 has a modest effect on increasing the absolute cell number during T cell development [18,21]. These studies support our view that exogenously added cytokine dose has limited effects on T cell development.

While the T cell development potential such as occurrence of CD8 ISP and DP cells were comparable for both culture systems, some differences exist, such as CD3 expression and development of TCRγδ cells. Cell-free or cell-cell signaling of the cytokines may account for the differences in proliferation and differentiation of the two systems. Nevertheless, differentiation to DP stage was inefficient and neither system supported terminal T cell maturation. Under both culture conditions, precursor proliferation rate declined beyond 30–35 days suggesting a discontinued dependence for IL-7 and Notch signals, consistent with previous reports [39,40], as such, this culture system alone does not support continued differentiation of adult human T cell precursors to CD3 and TCRαβ-bearing DP cells.

Signaling through IL-7/IL-7R supports survival and proliferation through DN3 stage in murine T cell development and the same is true for human T cell development [20,41]. In transgenic mice, expression of IL-7 under the control of *lck* promoter at low levels enhances proliferation of developing αβ T cells, but at high levels, it reduces proliferation and displays a marked block in DP transition [21]. Recent studies further support that IL-7R signals impair differentiation of CD8 ISP to DP cells in Zap70−/− and IL-7Rα transgenic mice [22], and IL-7R signals inhibit the expression of HMG domain transcription factors TCF-1, LEF-1 and RORγt, factors important for pre-T to DP transition [22]. In addition, IL-7 suppresses anti-CD3 antibody induced differentiation to DP stage in fetal thymus organ culture of Rag1-deficient mice [19]. Thus, we hypothesized that IL-7 withdrawal prior to ISP might be necessary for efficient DP transition. While IL-7 has been reported to display an inhibitory role in DP transition in murine T cell development, our results showed that the intermittent removal of IL-7 in the *in vitro* co-culture only had a minimal effect on human T cell DP transition.

The mechanism by which IL-7 inhibits T cell development is unclear. We observed abundant transcripts of TCF and LEF in T cell precursors at various time points. Thus, it seems unlikely that IL-7 withdrawal promotes T cell development by de-repressing transcription of the above factors. Our data suggest that IL-7 does not inhibit TREC formation, neither does it directly inhibit pre-TCR signaling. Interestingly, an increase in CD4 surface expression post IL-7 withdrawal may play a role in how these cells respond to anti-CD3 stimulation. As in human T cell development, CD4ISP precede DP stage, it is possible that increased CD4 may account for increased responsiveness to anti-CD3 stimulation. Alternative possibilities are, IL-7 mediates its effect through STAT-5 on transcription of genes necessary for pre-TCR expression and function, directly inhibits pre-TCR activation, or interferes with the TGFβ signaling pathway [42]. Detailed evaluation of these possibilities requires further investigation.

IL-7 mediates survival and proliferation of DN thymocytes [43]. In addition, IL-7 is required for TCRγδ gene rearrangement and also induces TCRβ chain rearrangement [44-46]. In order to progress to the next DP maturation stage, DN/ISP thymocytes must seize the rearrangement and express a functional TCRγδ or TCRαβ [17]. It is known that signaling via a functional TCR mediates allelic exclusion, survival and progression to SP stage [27]. Interestingly, in mice IL-7 signaling is inhibited at DP stage by down-regulating the IL-7Rα. In humans, IL-7R is expressed but its binding partner γC is down-regulated and STAT-5 responsiveness is lost [47]. Hence it is tempting to speculate that IL-7 signaling down-regulation might be an additional way of terminating rearrangement and preventing survival of T cells with non-functional TCRs. As both IL-7 and TCR signaling deliver survival signals, the down-regulation of IL-7 signaling ensures shutdown of an alternative survival pathway and selects for cells that respond to TCR signals. Clearly, the change in IL-7/IL-7R signaling is physiologically important and the reason for such regulation might reside on the intracellular signaling of IL-7/IL-7R on T cell activation and fate decision: proliferation, death or differentiation.

During T cell development, the appearance of ISP is dominated by CD4 ISP in human and CD8 ISP in mouse; pre-TCR signals drive proliferation, TCRα rearrangement, followed by the appearance of CD8 ISP in mouse and CD4 ISP in human. Interestingly, we observed CD8 ISP derived from human CD34 HPCs *in vitro*; we found a lack of proliferative burst and minimal rearrangement in the TCRα locus. Thus, the CD8 ISP may not be true ISP generated by pre-TCR signals, rather a result of cytokine-mediated CD8 expression [48].

**Figure 9:**
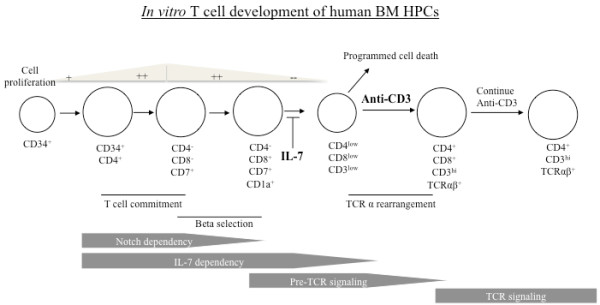
**Schematic illustration of*in vitro*T cell developmental program of adult human CD34**^**+**^**BM HPCs.** The key events, known surface markers and critical checkpoints of the *in vitro* developing T cells from adult human BM CD34^+^ HPCs under the stromal cell co-culture condition are illustrated.

T cell development is a complex process that involves multiple checkpoints and three-dimensional architecture composed of multiple cell types and compartments [49-52]. Our results, as summarized in Figure 9, demonstrate that signaling via CD3 and cell-cell contact was sufficient to drive differentiation to DP and subsequent CD4 lineage commitment in the absence of thymic environment. As OP9 cells do not express class II MHC molecules but the developing human thymocytes do (data not shown), the resulting CD4 cells are most likely selected by self MHC of the developing thymocytes. This alternate pathway of CD4 T cell development has been reported previously, especially in human T cell development [53,54]. Our study is in agreement with recent reports demonstrating that human HPCs when transplanted into immune compromised mice, can develop into CD4 T cells [55-58]. Such T cell development can occur via thymocyte-mediated selection, and does not require the presence of professional antigen presenting cells or epithelial cells expressing MHC-II. The human CD4 T cells derived from NOD/SCID γC^−/−^ mice receiving transplanted human HPCs display diverse v beta repertoire [56,57]. However, we found reduced diversity under the *in vitro* system illustrated by highly skewed vβ repertoire in most occasions. The three-dimensional environment *in vivo*, and the HLA expression on thymocytes may account for such differences. It is possible that TCRβ rearrangement and β selection events are inefficient in this system and the rare cells with properly arranged TCRs are selected upon anti-CD3 stimulation. Although one could challenge that expansion of contaminating T cells in the initial source of BM CD34^+^ HPCs may occur, we do not think this is possible as the fold increase post anti-CD3 stimulation would have been in a magnitude of over 300 fold in two weeks and we should also observed expansion of CD8 T cells along with CD4 T cells. Thus, even though lineage commitment to CD4 T cells can be achieved independent of thymic microenvironment, the latter is required for establishing a balanced TCR repertoire, supporting negative selection as well as promoting CD8 lineage commitment. 

## Conclusions

IL-7 withdrawal is necessary but not sufficient for further differentiation to DP stage, and anti-CD3 stimulation plays a key role in inducing CD3^+^TCRαβ^+^DP transition and subsequent maturation to CD4 T cells. Our findings further advance the experimental system required for *in vitro* modeling of adult human T cell differentiation, and will help develop novel approaches toward generating functional T cells from adult HPCs.

## Methods

### Human CD34^+^ cells and cell lines

The adult BM CD34^+^ HPCs from normal donors were purchased from AllCell Inc. (San Mateo, CA, USA). Control PBMCs were obtained from Civitan Regional Blood Center Inc. (Gainesville, FL) reviewed and approved by University of Florida Health Science Center Institutional Review Board (#507-1997, UF IRB-01). All studies involving human subjects are conducted in accordance with the guidelines of the World Medical Association's Declaration of Helsinki (most recent revision). The mouse fetal stromal cells (OP9) were purchased from the American Type Culture Collection (ATCC, Manassas, VA) and maintained as previously described [34]. The engineered LmDL1 and LmDL1-FL7 cell lines were generated by transducing cells with lentivectors encoding DL1, Flt3L and IL-7, respectively. The stromal cells were maintained in α-MEM (Invitrogen/Gibco BRL, Grand Island, NY) supplemented with 15-20% fetal bovine serum (FBS, Invitrogen/Gibco BRL) and 1% Penicillin-Streptomycin (Mediatech Inc., Manassas, VA). IL-7 cytokine secretion was measured by using human IL-7 ELISA kit (Ray Biotech, Inc) and soluble Flt3L in culture was measured using human Flt3L ELISA kit (Assay Biotechnology Company, Inc, Sunnyvale, CA). Cell free supernatants were harvested from LmDL1 and LmDLFL7 cells cultured for 48 hrs (80-90% confluent), in a 12 well plate containing 1 ml of media. The samples were read on model 680 microplate reader (Bio-Rad). The surface expression of mouse DL1 and Flt3L was analyzed by flow cytometry with Alexa Fluor 647-conjugated anti-DL1 Ab (Biolegend) and purified anti-Flt3L Ab (Abcam Inc. Cambridge, MA) conjugated with Zenon-Alexa 488 according to manufacturer’s instructions (Invitrogen).

### LmDL1 stromal cell and CD34^+^ HPC co-culture

The CD34^+^ HPCs were seeded into 24-well-plate at 1x10^5^ cells/well containing a confluent monolayer of LmDL1 or LmDL1-FL7 cells. The cocultures were maintained in complete medium from day 1, consisting of α-MEM with 20% FBS and 1% Penicillin-Streptomycin, supplemented with 5 ng/ml IL-7 (PeproTech, Inc. Rocky Hill, NJ) and 5 ng/ml Flt3L (PeproTech, Inc.) as indicated. The cocultures were replenished with new media every 2–3 days. The cells in suspension were transferred to a new confluent stromal monolayer once the monolayer began to differentiate or when developing cells reached 80-90% confluent. The cells were transferred by vigorous pipetting, followed by filtering through a 70 μm filter (BD/Falcon, BD Biosciences, Sparks, MD) and centrifugation at 250 RCF, at room temperature for 10 min. The cell pellet was transferred to a fresh confluent monolayer. The cells were harvested at the indicated time points during the T cell development for analysis.

### Monoclonal antibodies and flow cytometry

The surface expression of mouse DL1 and Flt3L was analyzed by flow cytometry with Alexa Fluor 647-conjugated anti-DL1 antibody (Biolegend) and purified anti-Flt3L antibody (Abcam Inc. Cambridge, MA) conjugated with Zenon-Alexa 488 according to manufacturer’s instructions (Invitrogen). The antibodies used for surface staining of T cell development included CD4 (clone RPA-T4 Pacific blue), CD8 (clone RPA-T8 PE), CD3 (clone UCHT1, Pacific Blue, clone SK7, PE-Cy7), TCRαβ (clone T10B9.1A-31, FITC), CD1a (clone HI149, APC), CD7 (clone M-T701, FITC, PE), and intracellular staining for Ki67 (clone B56, FITC), and isotype IgG1κ, which were from BD Biosciences (San Jose, CA). anti-CD127 (clone 40131-FITC) was from R&D systems (Minneapolis, MN). Vβ repertoire analysis was performed using IOTest® Beta Mark TCR Vβ Repertoire Kit according to manufacturer’s instructions (Beckman Coulter, Fullerton, CA). For flow cytometric staining, cells were first washed with PBS plus 2% FBS and blocked with mouse and human serum at 4°C for 30 min. Cells were incubated with antibodies per manufacturer’s instructions. For each fluorochrome-labeled Ab used, appropriate isotype control was included. After antibody staining, the cells were washed twice and fixed with 2% para-formaldehyde. Intracellular staining was performed using BD cytofix/cytoperm kit, according to the manufacturer’s protocol. Data was acquired using BD FACS Diva software (version 5.0.1) on a BD FACSAria or a BD LSR and analyzed using the Flowjo software (version 7.1.3.0, Tree Star, Inc. Pasadena, TX).

### T cell stimulation and effector function analysis

To stimulate naïve T cells, a protocol for long term stimulation was followed using anti-CD3/CD28 beads (Dynal/Invitrogen, San Diego, CA) per manufacturer’s instructions. The cells and the beads were mixed and plated into a 96 well plate at 37°C for 2–3 days in X-Vivo 20 (Gibco) media, on day 3, 12.5 U of IL-2, 5 ng/ml of IL-7 and 20 ng/ml of IL-15 were added and the cells were cultured for additional 11–12 days. The *in vitro* expanded CD4 T cells were stimulated with PMA and Ionomycin (Sigma-Aldrich, St. Louis, MO), and analyzed for the release of IFN-γ, IL-4 and IL-17. Briefly the cells were incubated with 25 ng/ml PMA and 1 μg/ml ionomycin for one hour followed by the addition of 6 μg/ml monensin (Sigma-Aldrich) to inhibit Golgi-mediated cytokine secretion. After 4–5 hours of incubation, the cells were harvested and surface stained for CD4 (clone RPA-T4, Pacific blue), CD8 (clone SK1, APC-Cy7), CD3 (clone SK7 PE-Cy7), CD25 (clone M-A251, PE) and intracellular stained for IFN-γ (clone 25723.11, FITC), IL-4 (clone MP425D2, APC), FOXP3 (clone PCH101, Alexa 647); the above antibodies were from BD Biosciences, and IL-17 (clone 64CAP17, PE) antibody was from e-Biosciences. The data were collected by flow cytometry using BD FACSAria and analyzed using Flowjo.

### RT-PCR

RNA was harvested from cells using TRI-Reagent (Sigma-Aldrich) and 1 ug RNA was reverse transcribed into cDNA by using Two-step AMV RT-PCR kit (Gene Choice, MD). The following primers were used for the PCR: mGAPDH, F (Forward) 5’-TCA CCA CCA TGG AGA AGG C-3’ and R (Reverse) 5’-GCT AAG CAG TTG GTG GTG CA-3’; mDL1, F 5’-GCT CTT CCC CTT GTT CTA ACG-3’ and R 5’-CAC ATT GTC CTC GCA GTA CC-3’; Flt3L, F 5’-AAG GAT CCG CAG GAT GAG GCC TTG-3’ and R 5’-CGG CGA CAG GAG GCA TGA G-3’; IL-7, F 5’-TTC TCG AGT TAT CAG TGT TCT TTA GTG-3’ and R 5’-AAG CGG CCG CCA CCA TGT TCC ATG TTT CT-3’; huGAPDH, F 5’-CCG ATG GCA AAT TCG ATG GC-3’ and R 5’-GAT GAC CCT TTT GGC TCC CC-3’; hLEF-1, F 5'-CGA CGC CAA AGG AAC ACT GAC ATC-3' and R 5'-GCA CGC AGA TAT GGG GGG AGA AA-3'; hTCF-1, F 5'-CGG GAC AGA GGA CCA TTA CAA CTA GAT CAA GGA G-3', and R 5'-CCA CCT GCC TCG GCC TGC CAA AGT-3'; Rag-1, F 5'-CAG CGT TTT GCT GAG CTC CT-3' and R 5'-GGC TTT CCA GAG AGT CCT C-3'; Rag-2 F 5'-GCA ACA TGG GAA ATG GAA CTG-3' and R 5'-GGT GTC AAA TTC ATC ATC ACC ATC-3'. After 30 cycles of amplification (95°C for 30 seconds, 55°C for 30 seconds, and 72°C for 60 seconds), PCR products were separated on a 2% agarose gel.

### T cell receptor excision circle (TREC) analysis

The TREC and RAG2 sequences were amplified by nested PCRs using two outer primers in the first round PCR, followed by adding two inner primers in the second round PCR using genomic DNA of PBMCs as templates, and cloned into pSTblue and verified by DNA sequencing. The two 5’ primers for TREC amplification were: outer 5'-AAT CTA GAG CAT GTT GCT TGA ACT CCT C-3', and inner 5'-AAT CTA GAG TAG CAT AAT TTC CTG GTT GAC-3'; the two 3’ primers for TREC amplification were: outer 5'-AAT CTA GAC CAA GGT GAA TCC TCT GAT C-3', and inner 5'-AAT CTA GAG TCC CAC ACT CCG TGC TG-3'. The two 5’ primers for RAG2 amplification were: outer 5'-AAG GAT CCA GCT GTG AAT TGC ACA GTC-3', and inner 5'-AAG GAT CCG CAA TCC TGA CTC AAA CTA AC-3'; the two 3’ primers for RAG2 amplification were: outer 5'-AAG GAT CCA GTT GAA TAG AAT GGT ACC-3’ and inner 5'-AAG GAT CCG TAA TCC AGT AGC CTG TCT C-3'. Cell lysates were prepared by proteinase K digestion (100 μg/mL) at 56°C for 1 hr, followed by heat inactivation at 95°C for 10 minutes. In brief, 1.5 μL of cell lysates equivalent to 100 ng DNA or 15,000 cells, were used as template for PCR amplification. The following primers were used for the PCR reaction for TREC: F 5’-CAG AGG GGT GCC TCT GTC A-3’ and R 5’-CTG TGA AAC ACT CCC CAG C-3’, and RAG2: F 5’-TCT TGG CAT ACC AGG AGA CA-3’ and R- 5’-AGT GGA ATC CCC TGG ATC TT-3’. PCR conditions were 95°C for 10 minutes, followed by 35 cycles of 95°C for 30 seconds, 55°C (RAG2) 59°C (TREC) for 60 seconds, 72°C for 60 seconds, with a final extension at 72°C for 10 minutes. PCR products were analyzed on 1% agarose gel.

### Statistical analysis

The statistical analysis was performed using Student’s *t*-test and GRAPHPAD PRISM 5 software.

## Competing interests

The authors declare no competing financial interests.

## Authors’ contributions

ESP and LJC designed the studies, carried out the experiments and drafted the manuscript; SO and KH performed the antibody staining and genomic PCR analyses; LJY, SKD and JSM participated in the design of the study; all authors read and approved the final manuscript.

## Supplementary Material

Additional file 1 **Optimized PCR condition for Rag2 and TREC genomic DNA amplification.***A,* Titration curves of cloned Rag2 and TREC templates of known concentrations. The standardization condition was applied to create a log dilution series for each assay. The coefficient of correlation for Rag2 were R = 0.9993, and for TREC R = 0.9992, indicating equal amplification of the templates over a range of input DNA concentrations. *B*, The equal and comparable slopes established for Rag2 and TREC PCR. Click here for file

Additional file 2 **Surface phenotype analysis of the*in vitro*differentiated CD4 T cells.** The T cells developed from adult BM HPCs in the LmDL1-FL7 co-culture following IL-7 withdrawal and anti-CD3/CD28 stimulation were analyzed for various surface markers as shown by flow cytometry, in comparison with control PBMCs. Click here for file
