# Supplementary figures and images for: Regulation of in vitro human T cell development through interleukin-7 deprivation and anti-CD3 stimulation
Source: BMC Immunol. 2012 Aug 16;13:46. doi: 10.1186/1471-2172-13-46 (PMC3496569; doi:10.1186/1471-2172-13-46)

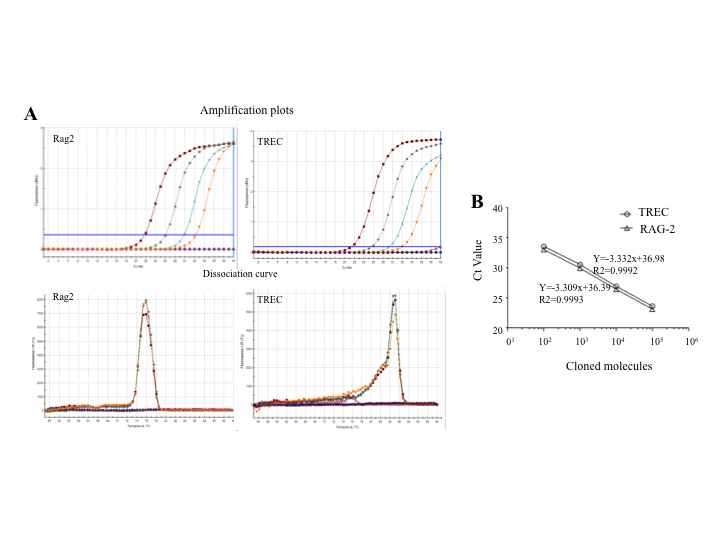

Supplement: Additional file 1 — Optimized PCR condition for Rag2 and TREC genomic DNA amplification.A, Titration curves of cloned Rag2 and TREC templates of known concentrations. The standardization condition was applied to create a log dilution series for each assay. The coefficient of correlation for Rag2 were R = 0.9993, and for TREC R = 0.9992, indicating equal amplification of the templates over a range of input DNA concentrations. B, The equal and comparable slopes established for Rag2 and TREC PCR. [file 1471-2172-13-46-S1.tiff]

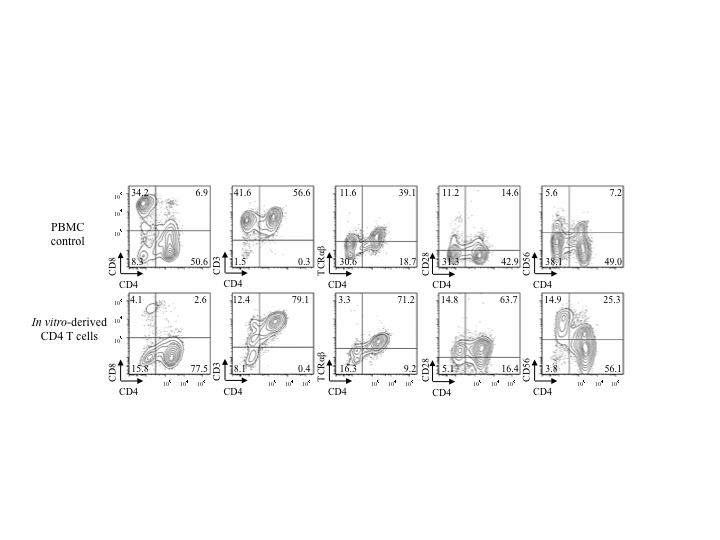

Supplement: Additional file 2 — Surface phenotype analysis of thein vitrodifferentiated CD4 T cells. The T cells developed from adult BM HPCs in the LmDL1-FL7 co-culture following IL-7 withdrawal and anti-CD3/CD28 stimulation were analyzed for various surface markers as shown by flow cytometry, in comparison with control PBMCs. [file 1471-2172-13-46-S2.tiff]
